# What Is the Role of Plastic Surgery in Global Health? A Review

**DOI:** 10.29252/wjps.7.3.275

**Published:** 2018-09

**Authors:** Mimi R Borrelli

**Affiliations:** King’s College London, WC2R 2LS, UK

**Keywords:** Plastic, Reconstructive, Surgery, Global, Health

## Abstract

There is growing awareness of the substantial global burden of surgical disease. Conditions treated effectively by plastic and reconstructive procedures make a large proportion of the global surgical diseases, and disproportionately affect individuals at the lower end of the economic spectrum. This article reviews the role of plastic surgery in global health, highlights the ongoing need for plastic and reconstructive surgery globally, and increasing efforts that have been made to meet these needs. There global shortage of plastic surgeons in low and middle income countries, but plastic surgery has a long tradition of humanitarian aid, has been a leader in global surgery development. Plastic and reconstructive surgical care has increasingly been shown to be cost effective and to have an immense impact on the economy of a region, delivering a substantial return on investment. More sustainable global surgical care is essential in future, requiring ongoing efforts from the plastic surgery community, greater recognition of the problems that can be addressed at policy level, and research to help guide policy-makers when facing the decision of allocating scarce resources. There is a fundamental role of plastic surgery in global health.

## Introduction

Global health is defined as ‘an area for study, research, and practice that places a priority on improving health and achieving health equity for all people worldwide’.^1^ There is growing awareness of the substantial global burden of surgical disease.^2^ Surgical conditions constitute 11% of worldwide disability-adjusted life years (DALYs).^3^ Around two billion people across the globe lack access to basic surgical services, and the burden is greatest in the poorest regions of the world.^4^

When services are accessed, their safety, timeliness, and effectiveness is often suboptimal, and patients may become financially ruined in the process.^4^^,^^5^ An additional four million healthcare workers^6^ and 143 million surgeries are needed annually in developing countries to avert death and disability.^4^ Plastic surgery is one surgical specialty gaining global momentum.^7^ A diversity of conditions across the world require plastic surgical intervention and they disproportionately affect people at the lower end of the economic spectrum, exacerbated by poverty and social circumstances.^8^ The global shortage of plastic surgeons in low and middle income countries (LMIC) to address this need is substantial.^8^ This essay discusses the global need for plastic surgical intervention and the increasing efforts that have been made to meet these needs, arguing for the fundamental role of plastic surgery in global health. 

*The Unmet Plastic Surgery Need*

Surgical conditions treated effectively by plastic and reconstructive procedures make a large proportion of the worldwide surgical disease burden. Sixty-six percent of the measured surgical disease DALYs are due to injuries, malignancy, or congenital anomalies, the three categories most frequently treated by plastic surgical intervention.^8^^,^^9^ Injuries and trauma comprise the greatest surgical burden.^10^ War, road-traffic accidents and natural disasters can injure soft tissue, tendons, nerves and bones, causing significant disability if untreated.^11^^,^^12^

**Fig. 1:**
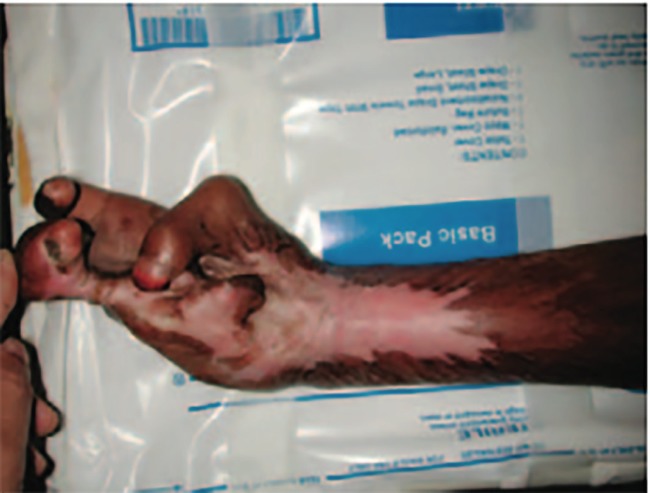
Severe electrical burn-related contracture of a young girl’s hand and forearm (16).

Simple, timely and inexpensive plastic surgical intervention from wound debridement, fracture fixation to soft tissue protection and closure, allow effective healing and reduce complications such as osteomyelitis and non-union, and avert permanent disability. Plastic surgical management of compartment syndrome following crush injuries, the second most common cause of death after earthquake, is crucially life-saving.^13^ Injuries rank as the third biggest health burden to LMIC^14^ and a lack of funding and health care infrastructure in LMIC means patients suffer disproportionately from the health consequences of war and natural disaster^15^ (Figure 1).

**Fig. 2:**
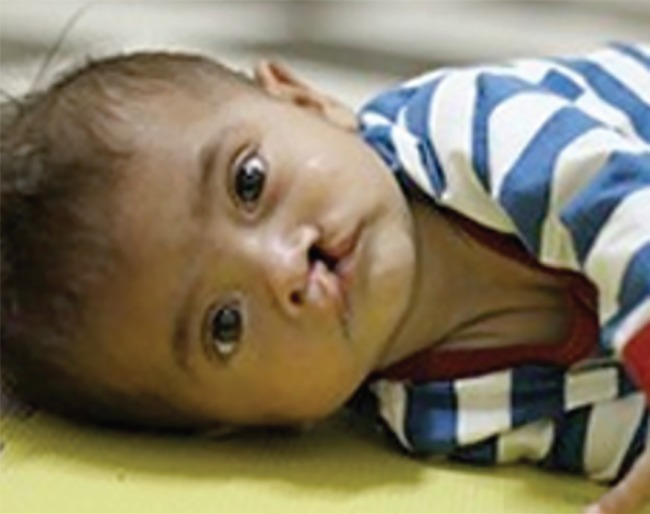
Child with cleft lip (20).

Worldwide, burns are responsible for a significant proportion of acquired deformities requiring reconstructive care.^2^^,^^16^ Around 10.9 million people suffer from severe burns each year.^17^ Burns are the 11th leading cause of death among children and the 5^th^ leading cause of non-fatal childhood injury.^18^ Burns are overrepresented in the poorest parts of the world, where large open flames are often used for cooking, livewires are exposed and fires result from destructive wars. Burns can cause extensive scarring, compromising form and function. They can lead to malnourishment, restricted mobility, sepsis, resulting in significant long-term disability.^19^ Many burns require plastic surgical expertise for burn excision, skin grafting, contracture release, corrective and reconstructive surgeries for optimal healing, as well as physiotherapy and splinting to help maintain function. This vast need for operative intervention is often not met in the developing world^17^^,^^20^ (Figure 1 and 2). 

Congenital anomalies account for an estimated 9% of the burden of surgical disease,^3^ and cleft lip and palate (CLP) are amongst the commonest congenital anomalies in the world, affecting 1 in 500-1000 live births^21^ (Figure 2). CLP increases perinatal mortality^22^ and surviving CLP infants are at increased risk of malnutrition, infection, and speech and feeding problems.^22^^,^^23^ Children with CLP may be stigmatized and ostracized from their communities, and be denied education and employment opportunities.^24^ Newborns with cleft deformities have been known to be suffocated by midwives.^24^

**Fig. 3:**
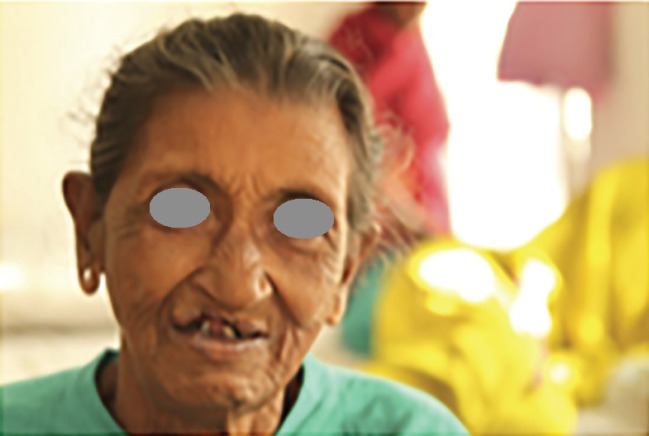
Elderly Indian lady with untreated cleft lip (30).

Lack of knowledge relating to CLP augments the problem in resource-limited countries.^25^ Dramatic improvement in appearance and speech can be achieved in one or two reconstructive operations, but in the developing world access to CLP care is severely limited. There is a backlog of around 4,000,000 untreated CLP patients worldwide,^26^ the majority of whom live in the developing world.^8^ These people live with the physical, psychological and socioeconomic consequences of clefts throughout their lives^27^^-^^29^ and cause a tremendous cost to productivity^27^^-^^30^ (Figure 3).

**Fig. 4:**
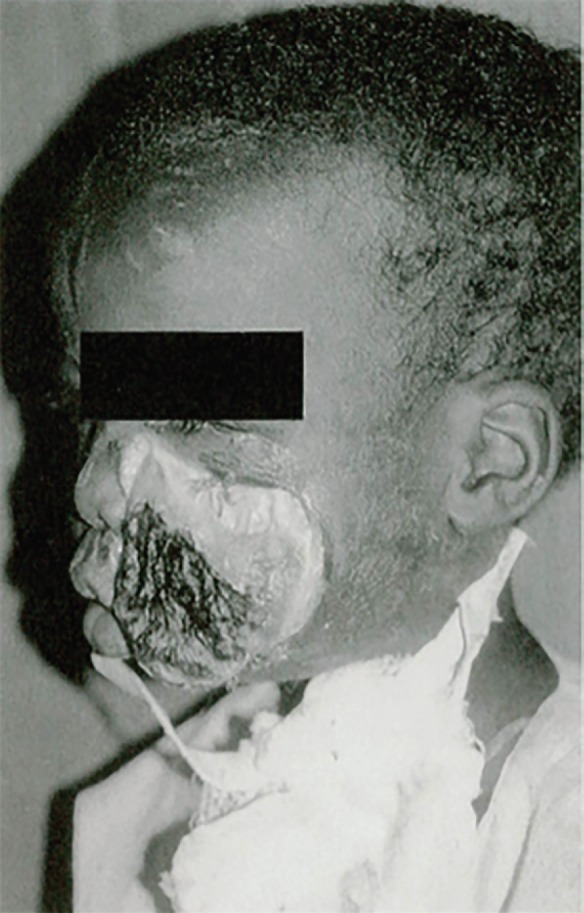
African child with noma of the left cheek (32).

There are a number of additional global diseases that lead to problems requiring a plastic and reconstructive intervention. Congenital diseases include syndactyly or constriction-band syndrome,^31^ and acquired conditions include surgical infections such as necrotizing soft tissue infections, abscesses, osteomyelitis. noma (necrotizing ulcerative stomatitis), an orofacial gangrene found mainly in children, is linked to poverty, malnutrition and disease.^32^ A sums of 140 000 patients worldwide are affected each year. The mortality rate is 90% and survivors often suffer from serious facial disfigurement, trismus, oral incontinence and speech problems (Figure 4).

**Fig. 5:**
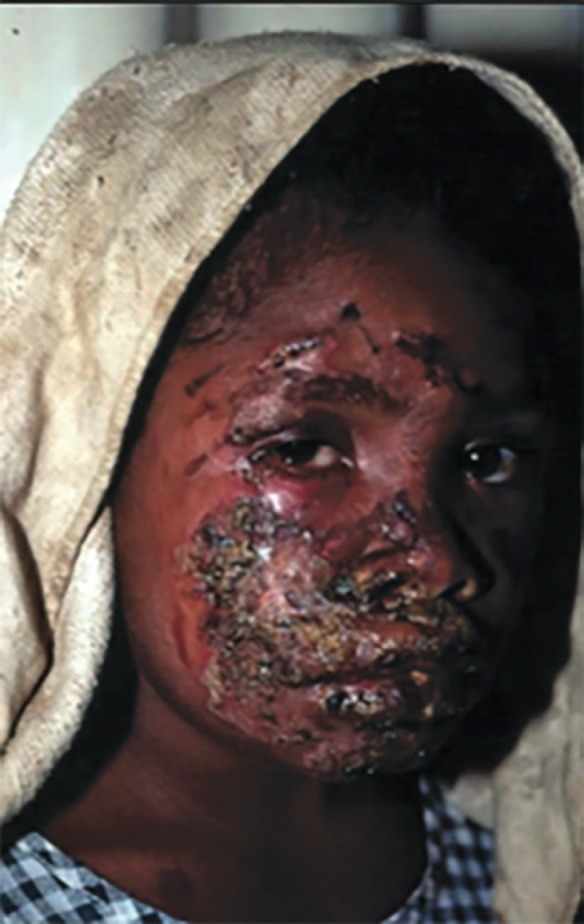
West African girl with facial burns from falling into the open kitchen fire during a seizure. Transport delay from her village to the health clinic meant she had superimposed infection and conjunctival injury at risk of cataracts at the time of presentation (36)

Plastic surgical services are recognized as relatively low-cost means of alleviating or resolving the immense suffering from these disabling conditions.^7^^,^^33^ Developing countries frequently lack resources devoted to plastic surgery to meet the demand.^10^ In China or India, there are less than one tenth of the plastic surgeons per million people as in North America (~1.5 *vs* ~6 surgeons per million).^8^ Countries in Sub-Saharan Africa have less and in some cases none at all.^8^ Plastic surgical expertise tends to be concentrated in urban areas where surgeons may use their skills for cosmetic purposes or other more lucrative interventions, rather than to decrease the burden of disease in the far more populous rural regions.^34^ It is clear that globally there are unmet plastic surgical needs^35^^,^^36^ (Figure 5). 

*Meeting the Need*

Action to improve health and health equity is integral to global health.^37^ Plastic and reconstructive surgery has a long tradition of international service and the humanitarian importance of the discipline is now stronger than ever.^38^ Over 100 plastic surgery non-profit associations have been founded, aiming to increase plastic surgical services for people in the developing world.^7^ ReSurg (previously Interplast), Operation Smile, and Smile Train are examples targeting safe and effective CLP care.

**Fig. 6:**
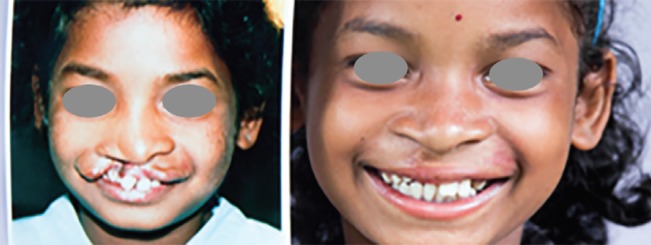
Cleft lip before and after repair (20).

Surgical “mission-trips” have been one approach to address the gap in plastic surgical care and increase the availability of plastic surgical care in LMIC. Missions are short-term humanitarian operations where human resources, expertise and supplies are delivered by a team of volunteer surgeons and medical personnel into areas of the world with limited access to specialized medicine.^39^^-^^41^ Humanitarian organizations have organized outreach programs for decades, and today hundreds of reconstructive and plastic surgery missions are conducted in the developing world providing surgeries for craniofacial deformities, congenital defects including CLP repair, burns, and trauma^42^^-^^44^ (Figure 6).

The ‘mission model’ has been immensely effective in delivering safe, well-timed, high-quality surgery to patients requiring reconstructive treatment in manners congruent with needs and constraints.^29^^,^^45^ Surgical missions affect immediate change that has sustained impact on health and quality of lives for patients, their families^29^ and their society.^27^ CLP surgical mission trips are highly cost-effective and compare favorably to other diseases targeted by global aid.^46^^,^^47^

Successful missions involve effective communication and balanced teams of technicians, nurses, anesthetists, administrators and occupational therapists.^17^ Partnerships between governments, national ministries of health, non-profit organizations and local medical personnel are essential to improve pre-mission patient identification and ensure pre- and postoperative care. This partnership is also necessary to manage country logistics and bureaucratics, and provide cultural and linguistic competency to complement the technical skills of the surgical team.^16^^,^^48^^,^^49^ Quality control protocols ensure delivery of safe, sustainable, ethically sound, high-quality care.^45^^,^^50^ Cultural awareness and sensitivities to patients’ social and cultural expectations are important in providing patient-centered care.^17^

**Fig. 7:**
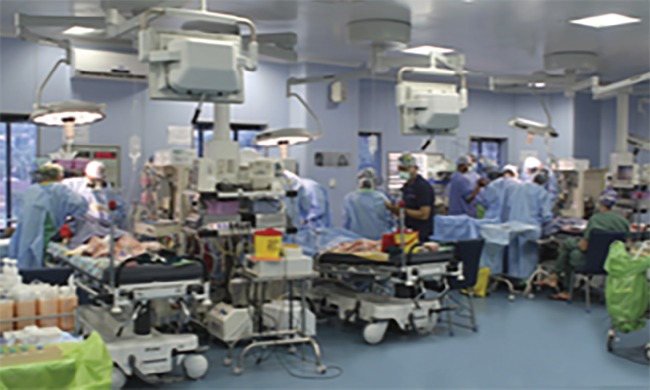
The operating suite of a state-of-the art surgical facility at a care center with an open layout, advanced surgical equipment, sophisticated anesthesia and monitoring capabilities (30).

In humanitarian plastic surgical initiatives there has been a shift from supporting missions to building care centers, which prioritize sustainability and build quantity, capacity and availability of the local health care systems through education or financial support.^29^^,^^30^ Developing healthcare infrastructure involves empowering medical staff, improving education and training of in-country personnel and construction of new units, such as cleft centers^51^ (Figure 7). Supporting local surgeons in their care for their indigent patients is the most cost-effective method of increasing access to care.^35^

Unlike missions none of the travel or similar costs associated with foreigners are incurred. Sustainable development creates long-term independent health systems and provides integrated care (e.g. physiotherapy and wound care) and long-term follow-up to augment plastic surgical services, enhance the status of local medical personnel and avoid service gaps in the wake of the mission.^7^^,^^40^ Developing the health system as a whole only has minimally distortive effects on local health care as compared to mission models, and may stimulate the local microeconomy.^30^ Multilateral partnerships between local and international teams are of primary importance in building sustainable surgical efforts in developing countries.^28^ Collaboration generates a gateway for cross-cultural education exchange and the introduction of new surgical practice techniques^17^ (Figure 7).

Education of healthcare personnel and students is fundamental to sustainability and necessary for responding to the global surgical workforce crisis.^52^ Education enables rapid dissemination of medical and surgical knowledge in LMIC, across a range of healthcare professions, covering a variety of topics from specific surgical operations to physiotherapy skills, nursing and post-anesthesia care.^22^ Training of local medical staff enables them to perform progressively more complex procedures and ultimately they become self-sufficient.^53^ This is more cost-effective and sustainable than importing foreign teams and can have a greater impact locally.^28^ Practitioners can be supported to train elsewhere if training is not available in their native country, but risks the educated, qualified practitioners not returning, contributing to the “brain drain” of plastic surgeons.^54^

**Fig. 8:**
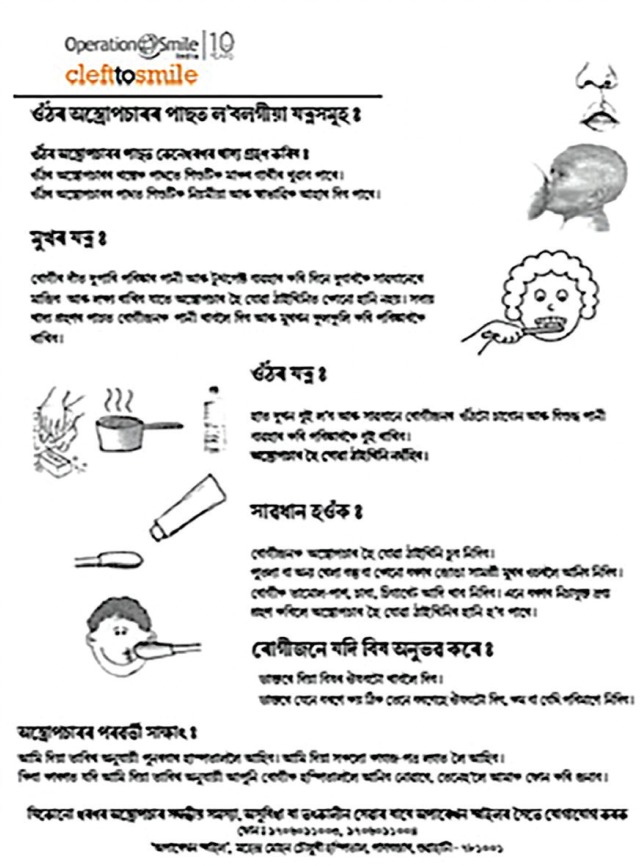
Discharge information for post-operative CLP patients written in the native language (Assamese) with pictographs for illiterate patients (55)

Efforts have also been made to educate patients. One group provided a discharge sheet to patients outlining postoperative cleft repair care (Figure 8). This significantly reduced the incidence of lip wound infections.^55^ For volunteers, international aid enhances cultural competency, fosters a deeper appreciation for global public health issues and hones the skills paramount in reducing the surgical burden of disease.^56^^,^^57^ It also makes it increasingly likely for that person to incorporate international volunteerism as part of their career.^58^ (Figure 8).

Both mission and care center models have their own unique advantages and are complementary rather than competing. Missions are better able to manage present emergent needs, and care models look to improve long-term gains. A center may be better placed to approach surgical care delivery than medical missions when the country’s geopolitical environment allows for a permanent health care presence.^59^ Care centers are more cost-effective than mission-based care,^59^ although both models are still highly cost-effective and worthy of global investment.^60^ Their mutual goals are to improve access to those who are deprived of plastic surgical intervention because of their circumstances and global location, and ultimately to improve outcomes and quality of life. 

There are a number of challenges associated with trying to increase access to plastic surgical care in the developing world. Political issues, including war and conflict can exacerbate unmet surgical needs. Threats of violence increase the risks for any program involving foreigners. Inadequate funding, financial means and insufficient sponsorships are on-going challenges that affect the long-term sustainability of even the most consistent projects and interventions.^40^^,^^61^

There is growing awareness of the immense worldwide burden of surgical disease. This essay has argued for the significant role of plastic surgery in global health. Many conditions are in need of practitioners with plastic surgical expertise, especially in LMIC.^8^ Plastic surgery has a long tradition of humanitarian aid and in many ways has been a leader in global surgery development in LMIC.^28^ Plastic and reconstructive surgical care has increasingly been shown to be cost effective and to have an immense impact on the economy of a region, delivering a substantial return on investment.^2^^,^^27^

Policy and finances pose on-going difficulties. A more sustainable global surgical care is essential in future. This requires not only continued efforts from the plastic surgery community but also greater recognition of the problems that can be addressed at policy level.^35^ Research is critical in guiding policy-makers when facing the decision of allocating scarce resources. Consequently, more concrete data must be compiled to determine better the burden of disease amenable to global plastic surgical interventions.

## CONFLICT OF INTEREST

The authors declare no conflict of interest.
